# Reproducible genomic DNA preparation from diverse crop species for molecular genetic applications

**DOI:** 10.1186/s13007-017-0255-6

**Published:** 2017-12-02

**Authors:** Kelvin T. Chiong, Mona B. Damaj, Carmen S. Padilla, Carlos A. Avila, Shankar R. Pant, Kranthi K. Mandadi, Ninfa R. Ramos, Denise V. Carvalho, T. Erik Mirkov

**Affiliations:** 1 Texas A&M AgriLife Research and Extension Center, 2415 East US Highway 83, Weslaco, TX 78596 USA; 2 Department of Horticultural Sciences, Texas A&M University, 2133 TAMU, College Station, TX 77843 USA; 3 Department of Plant Pathology and Microbiology, Texas A&M University, 2132 TAMU, College Station, TX 77843 USA; 4 Present Address: Department of Plant Pathology and Microbiology, Texas A&M University, 2132 TAMU, College Station, TX 77843 USA; 5 Present Address: FuturaGene Ltd, Av. Dr. José Lembo, #1010 Bairro, Jardim Bela Vista, Itapetininga, São Paulo Brazil

**Keywords:** Genomic DNA extraction, Simplified SDS method, Polyphenols, Polysaccharides, *Saccharum* spp. hybrids, *Oryza sativa*, *Citrus sinensis*, *Solanum lycopersicum*, *Solanum tuberosum*

## Abstract

**Background:**

Several high-throughput molecular genetic analyses rely on high-quality genomic DNA. Copurification of other molecules can negatively impact the functionality of plant DNA preparations employed in these procedures. Isolating DNA from agronomically important crops, such as sugarcane, rice, citrus, potato and tomato is a challenge due to the presence of high fiber, polysaccharides, or secondary metabolites. We present a simplified, rapid and reproducible SDS-based method that provides high-quality and -quantity of DNA from small amounts of leaf tissue, as required by the emerging biotechnology and molecular genetic applications.

**Results:**

We developed the TENS-CO method as a simplified SDS-based isolation procedure with sequential steps of purification to remove polysaccharides and polyphenols using 2-mercaptoethanol and potassium acetate, chloroform partitioning, and sodium acetate/ethanol precipitation to yield high-quantity and -quality DNA consistently from small amounts of tissue (0.15 g) for different plant species. The method is simplified and rapid in terms of requiring minimal manipulation, smaller extraction volume, reduced homogenization time (20 s) and DNA precipitation (one precipitation for 1 h). The method has been demonstrated to accelerate screening of large amounts of plant tissues from species that are rich in polysaccharides and secondary metabolites for Southern blot analysis of reporter gene overexpressing lines, pathogen detection by quantitative PCR, and genotyping of disease-resistant plants using marker-assisted selection.

**Conclusion:**

To facilitate molecular genetic studies in major agronomical crops, we have developed the TENS-CO method as a simple, rapid, reproducible and scalable protocol enabling efficient and robust isolation of high-quality and -quantity DNA from small amounts of tissue from sugarcane, rice, citrus, potato, and tomato, thereby reducing significantly the time and resources used for DNA isolation.

**Electronic supplementary material:**

The online version of this article (10.1186/s13007-017-0255-6) contains supplementary material, which is available to authorized users.

## Background

Preparation of high-quality genomic DNA is critical for biotechnology applications, as well as molecular genetic studies. Isolation of DNA from agronomically important crops, such as sugarcane, rice, citrus, potato and tomato remains a limiting step due to the presence of high polysaccharides, polyphenols, and other secondary metabolites [1–5]. Furthermore, levels of these compounds accumulate in plants under abiotic and biotic stresses, such as drought or pathogen infection [6, 7]. These metabolites tend to copurify with DNA, interfering with downstream applications, including Southern blot hybridization, marker-assisted polymorphism detection, next-generation sequencing, PCR sequencing, and bacterial artificial chromosome library construction [5, 8–11].

A growing number of methods exist for isolating DNA from tissues of plant species with a high content of polysaccharides and/or secondary metabolites. These methods mainly use detergent-based extraction buffers containing cetyltrimethylammonium bromide (CTAB) [12–16], sodium dodecyl sulfate (SDS) [17–20] and guanidine [21]. Some methods have included in the CTAB or SDS extraction buffer, sugars such as mannitol [22] or sorbitol [23, 24], antioxidants such as PVP-40 (polyvinylpyrrolidone; molecular weight, 40,000) [3, 5, 11, 25–30], or high salt [31, 32]. Most methods use phenol/chloroform for purification of the extracted DNA; while others add a purification step, by binding the extracted DNA to glass fiber coated plate wells [33] or to an anion exchange column [34].

Here, we describe a simple, rapid and scalable procedure for isolation of high-quality and -quantity DNA from sugarcane, rice, citrus, potato, and tomato leaf tissue. The procedure is a simplified SDS extraction method with sequential steps of purification to remove polysaccharides and polyphenols using 2-mercaptoethanol and potassium acetate, chloroform partitioning, and sodium acetate/ethanol precipitation. We achieved high yields and quality of DNA consistently from small amounts of tissue samples for these different plant species in a short period of time with this new isolation method.

## Methods

### Genetic constructs and plant transformation

The *gus* gene (*gusA*, 1.811 kilobase [kb]) was cloned into the binary vector pBIN34S carrying the selectable marker neomycin phosphotransferase gene, to produce pBIN34S:*GUS* [35] for citrus transformation. pBIN34S:*GUS* was transformed into sweet orange (*Citrus sinensis* L. cv. Hamlin) and potato (*Solanum tuberosum* L. cv. Atlantic) using *Agrobacterium tumefaciens* strain EHA105 [36] and seedling-derived epicotyl (citrus) and internodal (potato) segments [35].

The pCAMBIA1301 binary vector carrying the *SHDIR16*:*GUS* construct (*gus* gene under the control of the culm-regulated *Saccharum* spp. hybrids *dirigent16* promoter) [37] was used for transformation of rice (*Oryza sativa* subspecies *japonica*, cultivar Taipei 309), using embryo-derived calli and *A. tumefaciens* strain EHA105 [38].

The *SHDIR16*:*GUS* construct together with the *Ubi1*:*BAR*/pUC8 (pAHC20) plasmid containing the *bar* (bialaphos resistance) selectable marker (under the control of the maize *ubiquitin 1* promoter) [39], were introduced into embryonic callus established from young leaf bases and immature flowers of commercial sugarcane (*Saccharum* spp. hybrids, cv. CP72-1210) by particle gun bombardment, as described previously [40].

### Plant growth

Sugarcane, rice and sweet orange were grown in Redi-Earth mix (Scotts, Hope, AR) in a controlled-environment greenhouse (28 °C with 14-h-light/10-h-dark). Tomato and potato were grown in Sunshine MVP mix (Sun Gro Horticulture, Agwam, MA, USA) in a controlled-environment room with sodium lamps (24 °C with 14-h-light/10-h-dark).

### DNA isolation protocol

A simple and rapid protocol (TENS-CO), as described below, was developed for isolating DNA from sugarcane, rice, citrus, potato, and tomato tissues. The protocol steps are also outlined in Table 1.

**Table 1:** Major steps of the TENS-CO protocol used for plant genomic DNA isolation

(1) Extraction	TENS buffer^a^ and CO^b^
	5 M potassium acetate
(2) Precipitation	3 M sodium acetate (pH 5.2) (0.1× volume)
	Ethanol (2.0× volume)

^a^ TENS buffer: 100 mM Tris (hydroxymethyl) aminomethane (Tris Base) (pH 8.0), 50 mM ethylenediamine-tetraacetic acid (EDTA) (pH 8.0), 500 mM sodium chloride (NaCl), 1% (w/v) sodium dodecyl sulfate (SDS), and 2% (v/v) 2-mercaptoethanol

^b^ *CO* chloroform:octanol (24:1)

#### Isolation of DNA by the TENS-CO method

##### Extraction

Fresh tissue from young leaves (0.15 g) (snap frozen in liquid nitrogen, mostly used) was homogenized in 2 ml screw-cap microcentrifuge tubes for 20 s at 5000 rpm with the Precellys 24 homogenizer (MO BIO Laboratories, Carlsbad, CA, USA) in the presence of two steel BB air gun beads (BB refers to the bead size, 4.5 mm-diameter) (Walmart Supercenter, Bentonville, AR, USA). Up to 24 samples could be processed at a time with this homogenizer. Alternative to liquid nitrogen, microcentrifuge tubes with leaf samples were frozen at – 80 °C for at least 1 h before homogenizing. Extraction buffer (1.25 ml) was added to each homogenized leaf sample and mixed by vortexing. The extraction buffer consisted of 100 mM Tris (hydroxymethyl) aminomethane (Tris Base), 50 mM ethylenediamine-tetraacetic acid (EDTA) (pH 8.0), 500 mM sodium chloride (NaCl), 1% (w/v) sodium dodecyl sulfate (SDS), and 2% (v/v) 2-mercaptoethanol. The homogenate was incubated at 65 °C for 30 min and centrifuged at 10,000×*g* for 10 min. The collected supernatant (1.3 ml) was mixed with 0.4× volume of 5 M potassium acetate (KOAc) (520 µl), vigorously mixed by hand, and incubated on ice for 20 min. The mixture was centrifuged at 10,000×*g* for 15 min at 4 °C. The supernatant (1.4 ml) was collected and mixed with 0.5× volume of chloroform:octanol (24:1) (700 µl) vigorously by inversion for 10 min, and centrifuged at 13,000×*g* for 15 min. The upper aqueous phase (1.2 ml) was collected, mixed with 66 µg of RNase (6.6 µl of 10 mg/ml), and incubated in a 37 °C water bath for 1 h.

##### Precipitation

The mixture was added to 0.1× volume of 3 M sodium acetate (pH 5.2) and 2× volume of ice-cold 100% (v/v) ethanol and incubated at – 20 °C for 1 h. The DNA pellet was recovered by centrifugation at 12,000×*g* for 10 min at 4 °C, washed with 70% (v/v) ethanol, air-dried, and dissolved in 200–400 µl of nuclease-free water.

#### Comparative analysis of the TENS-CO method with standard isolation methods

An SDS-based method (modified from Tai and Tanksley [20]) and a CTAB-based method (modified from Chee et al. [41]) conventionally used for sugarcane and citrus (3.0 g of tissue), respectively, were tested in parallel with the TENS-CO method (0.15 g of tissue) for DNA extraction. Two commercial kits, PowerPlant Pro DNA Isolation Kit (SDS-based; MO BIO Laboratories; for rice, potato and tomato) (0.15 g of tissue) and Synergy Plant DNA Extraction Kit (CTAB-based; OPS Diagnostics, Lebanon, NJ, USA; for potato and tomato) (0.05 g of tissue) were also tested.

### DNA yield and purity

DNA was quantified by measuring the concentration and absorbance (A) level at 230, 260, and 280 nm using a NanoDrop 1000 Spectrophotometer (Thermo Fisher Scientific, Wilmington, DE, USA). The absorbance ratios of A_260:280_ and A_260:230_ were used to determine the purity of DNA. The quality of DNA was examined by electrophoresis on 0.8% agarose gels stained with ethidium bromide.

### Southern blot analysis

Genomic DNA was isolated from young leaves of 6-month-old transgenic sweet orange and potato plants, and 3–4 month-old transgenic sugarcane and rice plants. Genomic DNA (15 µg each lane) was digested overnight with either *Hind*III (sweet orange and potato) or *Sac*I (sugarcane and rice), electrophoresed on 0.8% (w/v) agarose gels and transferred to nylon membranes (Amersham Hybond-XL, GE Healthcare Bio-Sciences Corp., Piscataway, NJ, USA) in an alkaline solution (0.4 M sodium hydroxide) [42].

DNA blots were hybridized with the full-length coding region of *gusA* (2.28 kb). The *gusA*-specific probe was obtained from pAHC27 [39] by *BamH*I and *Sac*I digestion. Probes were labeled with [^32^P] α-dCTP by random priming using the Random Primers DNA Labeling kit (Invitrogen, Carlsbad, CA, USA). Pre-hybridization, hybridization, washing and detection of DNA gel blots were performed as described by Mangwende et al. [43], using Church’s buffer.

### Quantitative real-time PCR

DNA was isolated from leaves of 6 week-old healthy and *Candidatus* Liberibacter solanacearum (Lso) infected potato plants. Top-most fully expanded non-inoculated leaves were collected from healthy and Lso infected plants (10 psyllids per plant) at 21 days post infestation. Quantitative real-time PCR (qPCR) was used to detect Lso in infected and healthy samples with CL-ZC primers specific for detection of Lso [44] and *TOMATO RIBOSOMAL PROTEIN L2* (RPL2) (GenBank accession no. X64562) endogenous reference gene primers [45]. The primer sets for Lso (CL-ZC-F: 5′-ACCCTGAACCTCAATTTTACTGAC-3′ and CL-ZC-R: 5′-TCGGATTTAGGAGTGGGTAAGTGG-3′) and RPL2 (RPL2-F: 5′-GAGG-GCGTACTGAGAAACCA-3′; RPL2-R: 5′-CTTTTGTCCAGGAGGTGCAT-3′) were used to perform qPCR on a CFX384™ Real-Time PCR Detection System (Bio-Rad Laboratories, Inc., Hercules, CA, USA) with the i*Taq*™ universal SYBR^®^ Green supermix (Bio-Rad Laboratories, Inc.), 0.2 µM of target specific primer and 50 ng of genomic DNA, according to the manufacturer’s instructions. qPCR was performed on DNA from four biological samples with three technical replicates per sample using the following conditions: one cycle at 95 °C for 3 min, 39 two-step cycles each at 95 °C for 15 s and 55 °C for 30 s, and a final melting curve of 65–95 °C for 5 s. Results were analyzed and recorded as C_t_ (threshold cycle) values, and melt curve analysis was performed to detect non-specific amplification. Quantification of Lso in the infected versus healthy tissues was determined relative to that of the *RPL2* gene using the comparative C_t_ method (2^− ΔΔCt^) [46].

### Marker-assisted selection

The co-dominant Sequence Characterized Amplified Regions (SCAR) marker P6-25 linked to *Tomato yellow leaf curl virus* (TYLCV) resistance gene *Ty*-*3* [47] was used to select for resistant plants. DNA was isolated from leaves of segregating tomato lines at the Vegetable Breeding Program, Texas A&M AgriLife Research-Weslaco, Texas. PCR was performed on a C1000 Touch™ thermal cycler (Bio-Rad Laboratories, Inc.) in a total reaction volume of 25 µl using 100 ng of DNA, 0.2 µM of each target specific primer, 1.0 U of Platinum™ *Taq* DNA polymerase (Invitrogen), 1× PCR buffer, 1.5 mM of MgCl_2_ and 0.2 mM of each dNTP. PCR conditions were: one denaturing cycle at 94 °C for 4 min, 35 cycles each at 94 °C for 30 s, 53 °C for 1 min and 72 °C for 1 min, and a final extension cycle at 72 °C for 10 min. PCR amplicons were separated by electrophoresis on a 1.5% (w/v) agarose gel stained with ethidium bromide. Allele identification was performed based on fragment sizes as reported by Ji et al. [47].

## Results and discussion

### Preparation of high-quality and -quantity DNA by the TENS-CO method

**Fig. 1:**
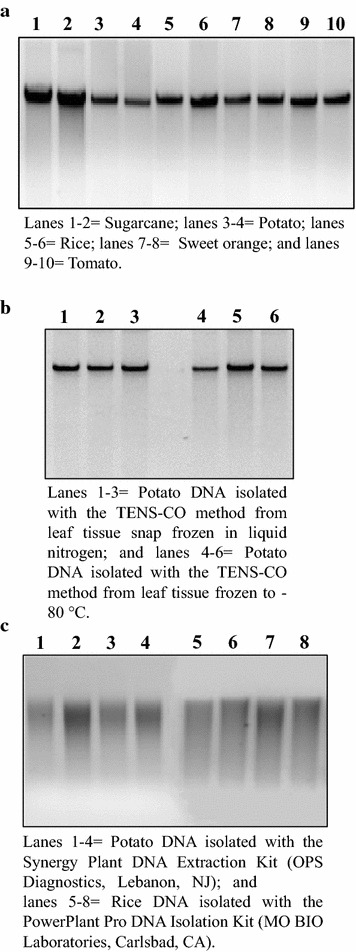
Assessment of the integrity of the genomic DNA isolated with the TENS-CO method by gel electrophoresis. DNA extracted from leaves of different plant species using TENS-CO after freezing (snap) in liquid nitrogen (**a**, **b**) or to – 80 °C (**b**) as well as two commercial DNA isolation kits (**c**) was electrophoresed on a 0.8% (w/v) agarose gel stained with ethidium bromide (approximately 300–400 ng per lane)

The TENS-CO method yielded DNA with high-purity and -integrity from liquid nitrogen and − 80 °C frozen tissue as reflected by the intact bands in gel electrophoresis (Fig. 1a, b) and low levels of polysaccharides/phenols as indicated by the spectrophotometric A_260_:A_230_ ratio values of 2.04, 2.18, 2.08, 2.23 and 2.10 for sugarcane, rice, sweet orange, potato, and tomato, respectively (Table 2). Ratio values above 1.9 are considered acceptable and indication of high-quality DNA. The presence of high amounts of NaCl (0.5 M) in the TENS-CO extraction buffer was efficient in increasing the solubility of polysaccharides, reducing their coprecipitation with the DNA in subsequent steps [48]. In addition, the incorporation of 2-mercaptoethanol into the extraction buffer helped in reducing phenolics oxidation and precipitation with DNA.

**Table 2:** Comparison of the TENS-CO method with standard DNA isolation procedures for the crop species used in the current study

Method	DNA yield (µg/mg tissue)	DNA purity	
		A_260_:A_230_	A_260_:A_280_
*Grass*			
Sugarcane (*Saccharum* spp. hybrids)			
TENS-CO	0.46 ± 0.05	2.04 ± 0.11	1.85 ± 0.01
Standard SDS (modified from Tai and Tanksley [20])	0.10 ± 0.01	1.61 ± 0.07	1.96 ± 0.02
*Cereal*			
Rice (*Oryza sativa* L. sp. *japonica*)			
TENS-CO	0.35 ± 0.05	2.18 ± 0.07	1.93 ± 0.01
MO BIO PowerPlant Pro Kit	0.10 ± 0.01	2.02 ± 0.05	1.84 ± 0.01
*Citrus*			
Sweet orange (*Citrus sinensis* L. cv. Hamlin)			
TENS-CO	0.64 ± 0.08	2.08 ± 0.04	2.00 ± 0.01
Standard CTAB (modified from Chee et al. [43])	0.12 ± 0.01	2.32 ± 0.09	1.92 ± 0.02
*Vegetables*			
Potato (*Solanum tuberosum* L. cv. Atlantic)			
TENS-CO	0.50 ± 0.04	2.23 ± 0.006	1.91 ± 0.04
MO BIO PowerPlant Pro Kit	0.05 ± 0.004	2.41 ± 0.05	1.94 ± 0.03
Synergy Plant DNA Kit	0.20 ± 0.01	2.18 ± 0.05	1.93 ± 0.02
Tomato (*Solanum lycopersicum* L. cv. Lance)			
TENS-CO	0.66 ± 0.06	2.10 ± 0.05	1.93 ± 0.01
MO BIO PowerPlant Pro Kit	0.05 ± 0.004	1.87 ± 0.08	1.80 ± 0.01
Synergy Plant DNA Kit	0.11 ± 0.01	1.93 ± 0.03	1.81 ± 0.01

*CTAB* cetyltrimethylammonium bromide and *SDS* sodium dodecyl sulfate

The TENS-CO method was also efficient in removing proteins, with DNA samples repeatedly exhibiting A_260_:A_280_ ratio values of 1.85 for sugarcane, 1.93 for rice, 2.00 for sweet orange, 1.91 for potato, and 1.93 for tomato (Table 2). The A_260_:A_230_ and A_260_:A_280_ ratios for MO BIO PowerPlant Pro DNA Isolation and Synergy Plant DNA kits were acceptable (Table 2), but the low yield restricts the applicability of the isolated DNA (Table 2). The incorporation of high concentrations of KOAc (5 M) aided in neutralizing the lysis produced by the TENS-CO buffer reaction and precipitating proteins that bind to SDS, thereby eliminating excess of proteins that form in the pellet after centrifugation.

The TENS-CO method produced good yields of DNA, equally from liquid nitrogen and − 80 °C frozen tissue, for sugarcane (0.46 ± 0.05 µg/mg), rice (0.35 ± 0.05 µg/mg), sweet orange (0.64 ± 0.08 µg/mg), potato (0.50 ± 0.04 µg/mg), and tomato (0.66 ± 0.06 µg/mg) (Table 2). The DNA concentrations were significantly higher than those obtained with the high-volume detergent-based standard isolation methods containing SDS (sugarcane, 0.10 µ/mg) and CTAB (sweet orange, 0.12 µg/mg) (Table 2). The commercial kits yielded low amounts of DNA for all species tested, i.e. rice (0.10 µg/mg for the SDS-based MO BIO PowerPlant Pro DNA isolation kit), potato (0.05 µg/mg for the MO BIO kit and 0.2 µg/mg for the CTAB-based Synergy Plant DNA kit), and tomato (0.05 µg/mg for the MO BIO kit and 0.11 µg/mg for the Synergy Plant DNA kit) (Table 2); furthermore, there was a mild degradation of the DNA as a result of the harsh homogenization suggested in the manufacturer’s protocols (Fig. 1c).

The TENS-CO method also resulted in higher yields of DNA from sugarcane than those reported by Vaze et al. [49] (0.025–0.1 µg/mg), and Honeycutt et al. [50] (0.28 µg/mg) (Additional file 1: Table S1). Similar results were observed for citrus and tomato DNA yields, which were less than 0.15 and 0.08 µg/mg, respectively (Additional file 1: Table S1) [4, 10, 51].

Optimization of the TENS-CO isolation method was achieved by reducing the leaf tissue weight from 0.4 to 3.0 g usually required for standard isolation methods for sugarcane, rice, citrus, potato and tomato to 0.15. Automated tissue homogenization allowed the extraction of 24 samples in a very short period of time (20 s) in small tubes (2 ml), using a reduced extraction volume (1.25 ml). High-throughput extractions could be achieved with TENS-CO by using higher capacity homogenizers.

The TENS-CO method is a simplified DNA isolation technique that uses SDS and an antioxidant (2-mercaptoethanol) for extraction and one precipitation step (ethanol) to yield high-quantity and -quality DNA from small amounts of tissue (0.15 g). The method is simplified and rapid in terms of requiring minimal manipulation, smaller extraction volume, reduced time needed for tissue homogenization (20 s) and DNA precipitation (one precipitation for 1 h). The procedure is scalable and can be used to study a larger number of samples.

### DNA isolated with the TENS-CO method is suitable for Southern blot analysis

**Fig. 2:**
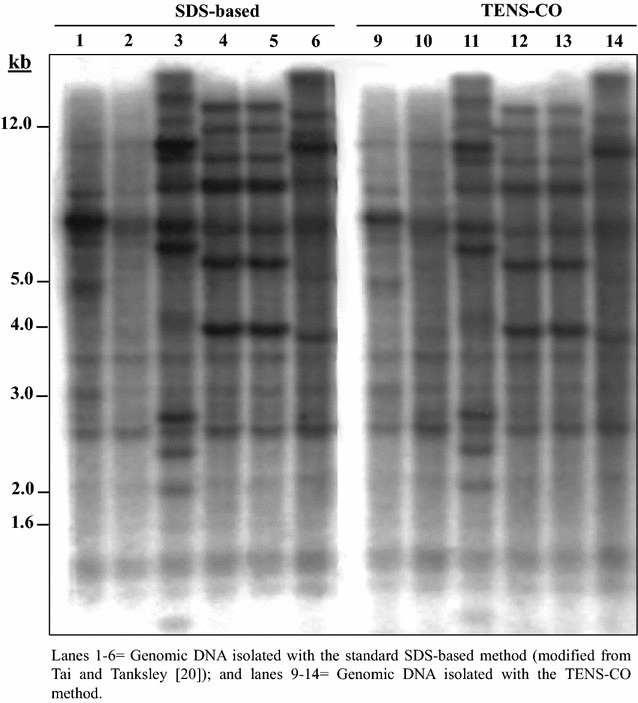
Southern blot analysis of *Hind*III-digested genomic DNA of representative sugarcane lines overexpressing the *gusA* reporter gene hybridized with a ^32^P-labeled DNA probe specific for *gusA*. DNA (15 µg each lane) was digested, blotted, hybridized, washed and imaged as described in methods

**Fig. 3:**
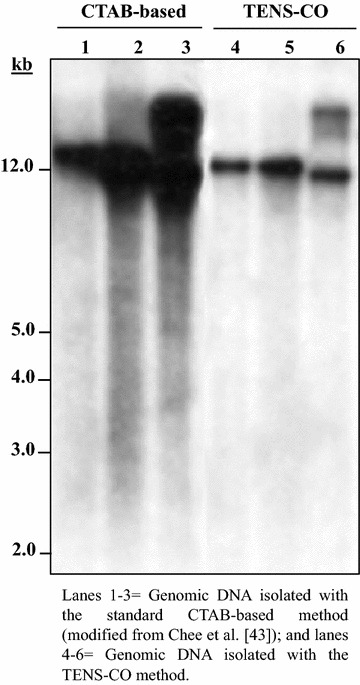
Southern blot analysis of *Sac*I-digested genomic DNA of representative citrus (sweet orange) lines overexpressing the *gusA* reporter gene hybridized with a ^32^P-labeled DNA probe specific for *gusA*. DNA (15 µg each lane) was digested, blotted, hybridized, washed and imaged as described in methods

Southern blot analysis is a robust technique widely used for molecular characterization of transgenics. A total of 11 sugarcane, 17 rice, six sweet orange and seven potato transgenic lines constitutively expressing the *gusA* reporter gene were used in this study. To demonstrate the suitability of the TENS-CO method for detecting specific genes, we determined the presence of the *gusA* transgene in transgenic sugarcane, rice, sweet orange and potato lines using Southern blot hybridization. Sugarcane and sweet orange genomic DNA, isolated by the TENS-CO and the standard methods (SDS- and CTAB-based methods for sugarcane and sweet orange, respectively) was digested with *Hind*III and *Sac*I, respectively and subsequently hybridized with the *gusA* probe. Transgenic sugarcane (Fig. 2) and sweet orange (Fig. 3) lines exhibited similar profiles of DNA isolated with the TENS-CO and standard methods. Binding of the *gusA* probe was as specific with DNA extracted by the TENS-CO as the one isolated with the standard methods (Figs. 2, 3, respectively), indicating that the DNA is intact. Furthermore, DNA extracted from the rice and potato transgenic lines using the TENS-CO and the traditional SDS- and CTAB-based methods displayed the same DNA hybridization profiles (data not shown). This indicates that the DNA isolated with the TENS-CO method is reproducible and can be used for screening a large number of transgenic lines of diverse plant species with high polysaccharides and secondary metabolites.

### DNA isolated with the TENS-CO method is successfully used for molecular diagnostics and pathogen detection

**Fig. 4:**
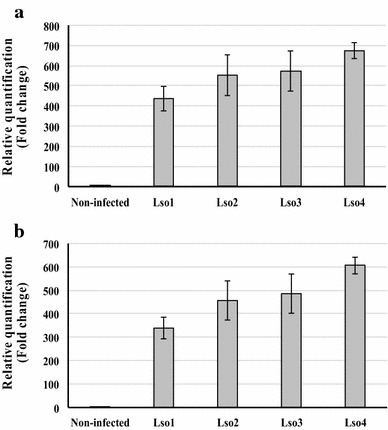
Quantification of the Zebra chip bacterium *Candidatus* Liberibacter solanacearum (Lso) in potato using quantitative PCR. Lso is detected in leaf DNA extracted with the TENS-CO method (**a**) or the standard CTAB-based method (modified from Chee et al. [43]) (**b**). Error bars indicate standard error of four biological replicates and at least three technical replicates

*Candidatus* Liberibacter solanacearum (Lso), an unculturable, phloem-limited bacterium transmitted by the potato/tomato psyllid *Bactericera cockerelli*, causes Zebra chip disease, which is a serious threat to potato and other Solanaceous crops, leading to significant economic losses in Central America, Mexico and the United States [52–54]. To date, no Lso-resistant varieties of potato or tomato have been reported. Recent control strategies of Zebra chip disease involved the use of insecticides to control psyllid populations in Lso-infected fields. Early detection of Lso in host leaves is crucial to strategies for the containment of the disease in its early stages, and qPCR is commonly used for this purpose [53–55]. The sensitivity and specificity of qPCR to detect pathogen in host tissue is dependent on various factors, including the quality of DNA. We tested the quality of DNA extracted by the TENS-CO method, as compared to the standard CTAB method for the detection of the Lso titer present in leaves of four potato plants infected with Lso (Lso1, Lso2, Lso3 and Lso4) using qPCR analysis. The qPCR results (from four biological samples and three technical replicates per sample) showed the presence of higher bacterial levels in all four Lso-infected potato samples as compared to non-infected ones, using DNA isolated with both TENS-CO and standard CTAB methods (Fig. 4a, b). The Lso levels detected with DNA from both methods were comparable, i.e. they were 434.5 (TENS-CO) or 338.6 (CTAB), 552.6 (TENS-CO) or 456.8 (CTAB), 573.6 (TENS-CO) or 486.8 (CTAB) and 675.6 (TENS-CO) or 606.8 (CTAB) fold more in Lso1, Lso2, Lso3 and Lso4 potato plants than in non-infected ones, respectively (Fig. 4a and b). The detection of different levels of Lso in all tested samples indicates that the TENS-CO DNA is of high quality to generate reproducible detection of microbial pathogens in host tissue.

### DNA isolated with the TENS-CO method is successfully used for marker-assisted selection

Crop cultivar development can be accelerated by the utilization of molecular tools that can reduce the time and cost of screening plants with desired traits. Marker-assisted selection has been successfully used in the selection of several specific genes/alleles for crop improvement to trace favorable alleles, pyramid desired genes into one genetic background, eliminate unwanted traits by breaking undesirable linkage, and increase the frequency of desired alleles in early segregating populations [56].

**Fig. 5:**
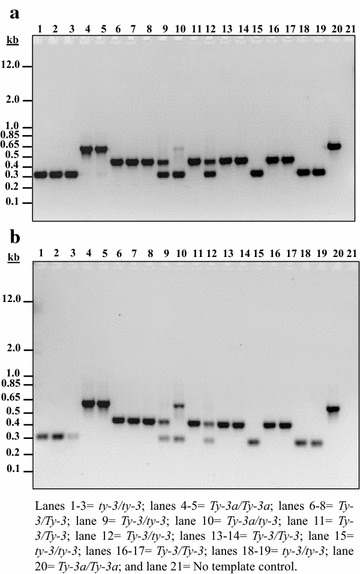
Marker-assisted selection using the co-dominant SCAR marker P6-25 linked to *Ty3* resistance gene in tomato [57]. PCR amplicons from DNA extracted with the TENS-CO method (**a**) or the standard CTAB-based method (modified from Chee et al. [43]) (**b**); were separated by electrophoresis on a 1.5% (w/v) agarose gel

A major disease affecting tomato production worldwide, especially in tropical and subtropical areas is TYLCV, vectored by whiteflies (*Bemisia tabaci*). Once tomato plants are infected, leaves curl upward and show strong crumpling, interveinal, and marginal yellowing, negatively impacting yield. Several major resistance genes for TYLCV have been identified, including the *Ty*-*3* gene from tomato wild-relative *S. chilense* [57]; however, this gene needs to be introgressed and stacked into locally adapted cultivars to ensure long-lasting disease resistance. Since marker-assisted selection requires genotyping large segregating populations, nucleic acid isolation methods that are simple, rapid and efficient will be useful. In order to test the TENS-CO method for marker-assisted breeding purposes, tomato plants from F_2_ segregating populations carrying the *Ty*-*3* resistance gene were evaluated for the presence or absence of the linked co-dominant SCAR marker P6-25. PCR amplification using DNA extracted with the TENS-CO method resulted in distinct P6-25 polymorphic amplicons linked to the *Ty*-*3* resistance gene (Fig. 5a). The amplicons obtained with DNA from the TENS-CO method were of the same size but of a higher intensity than those generated with DNA from the standard CTAB method (Fig. 5a, b). The 450-base pair (bp) and 650-bp fragments are linked to *Ty*-*3* resistance locus introgressed from *S. chilense* accessions LA2779 (*Ty*-*3*) and LA1932 (*Ty*-*3a*), respectively; while the 320-bp fragment corresponded to the sequence of the cultivated susceptible *S. lycopersicum* (*ty*-*3*) [57]. By this procedure, resistant homozygous and heterozygous plants can be distinguished from susceptible homozygous plants prior to field evaluation. DNA extraction with the TENS-CO method is cost effective, time saving, and amenable for semi-automated genotyping to screen large populations using conventional molecular laboratory equipment as part of a marker-assisted selection program.

## Conclusions

Genomic DNA isolation procedures have improved drastically over the past decade and are a powerful tool in molecular genetic and genomic research. To facilitate molecular genetic studies in major agronomic crops, we have developed a simple, rapid, reproducible and scalable protocol enabling efficient and robust isolation of high-quality and -quantity DNA from sugarcane, rice, citrus, potato, and tomato, thereby reducing significantly the time and resources used for DNA isolation. Compared to other protocols, the TENS-CO method is a simplified method consisting of one extraction step, using SDS and chloroform with 2-mercaptoethanol as an antioxidant, and one step of precipitation (sodium acetate/ethanol). We have demonstrated that the method accelerates screening of large amounts of plant tissues from species that are rich in polysaccharides and secondary metabolites for Southern blot analysis, marker-assisted selection and quantitative PCR applications.

## Additional file

**Additional file 1: Table S1.** Comparison of the TENS-CO method with other DNA isolation procedures used for plant species with high level of secondary metabolites. 

## Abbreviations

BAR: bialaphos resistance
C_T_: threshold cycle
CTAB: cetyltrimethylammonium bromide
EDTA: ethylenediamine-tetraacetic acid
GUS: β-glucuronidase
KOAc: potassium acetate
Lso: *Candidatus* Liberibacter solanacearum
NaCl: sodium chloride
PVP: polyvinylpyrrolidone
qPCR: quantitative polymerase chain reaction
RPL2: *tomato ribosomal protein L2*
SCAR: sequence characterized amplified regions
*SHDIR16*: *Saccharum* spp. hybrids *dirigent16*
SDS: sodium dodecyl sulfate
TYLCV: *tomato yellow leaf curl virus*
*Ubi1*: maize *ubiquitin 1*
