# Supplementary material for: Reproducible genomic DNA preparation from diverse crop species for molecular genetic applications
Source: Plant Methods. 2017 Dec 2;13:106. doi: 10.1186/s13007-017-0255-6 (PMC5712126; doi:10.1186/s13007-017-0255-6)
Supplement: Supplementary file 1 — Additional file 1: Table S1. Comparison of the TENS-CO method with other DNA isolation procedures used for plant species with high level of secondary metabolites. [file 13007_2017_255_MOESM1_ESM.docx]

**Table S1** **Comparison of the TENS-CO method with other DNA isolation procedures used for plant species with high level of secondary metabolites**

| **Method** | **DNA yield**  **(µg/mg tissue)** | **DNA purity** | |
| --- | --- | --- | --- |
|  |  | **A_260_:A_230_** | **A_260_:A_280_** |

***Grass***

Sugarcane (*Saccharum* spp. hybrids) *(present study)*

| TENS-CO (present study)  Standard SDS (modification, [1]) (present study)  CTAB/PVP [2] | 0.46 ± 0.05  0.10 ± 0.01  0.50 – 0.80 | 2.04 ± 0.11  1.61 ± 0.07  ND | 1.85 ± 0.01  1.96 ± 0.02  1.76 – 1.96 |
| --- | --- | --- | --- |

Sugarcane (*Saccharum officinarum*)

| CTAB/PVP [3]  CTAB/PVP [4]  CTAB/SDS [5] | 0.50  0.025 – 0.1  0.28 ± 0.16 | 2.00  1.7 – 1.8  ND | 1.93  ND  1.74 ± 0.18 |
| --- | --- | --- | --- |

Buffalograss (*Buchloe dactyloides)*

| SDS/CTAB/PVP 40 [6] | 0.27 | ND | 1.97 – 2.04 |
| --- | --- | --- | --- |

***Cereals***

Rice (*Oryza sativa* L. sp. *japonica*) *(present study)*

| TENS-CO (present study)  MO BIO PowerPlant Pro Kit (present study)  No detergent [7] | 0.35 ± 0.05  0.10 ± 0.01  0.12 ± 0.23 | 2.18 ± 0.07  2.02 ± 0.05  ND | 1.93 ± 0.01  1.84 ± 0.01  1.87 ± 0.05 |
| --- | --- | --- | --- |

Wheat (*Triticum aestivum*)

| SDS [8] | 0.50 – 0.80 | ND | > 1.77 |
| --- | --- | --- | --- |

Barley (*Hordeum sativum*)

| SDS [8]  SDS/PVP [9] | 0.50 – 0.80  0.02 – 0.10 | ND  ND | > 1.77  1.65 – 1.8 |
| --- | --- | --- | --- |

***Citrus***

Sweet Orange (*Citrus sinensis* L. cv. Hamlin) *(present study)*

| TENS-CO (present study)  Standard CTAB (modification, [10]) (present study) | 0.64 ± 0.08  0.12 ± 0.01 | 2.08 ± 0.04  2.32 ± 0.09 | 2.00 ± 0.01  1.92 ± 0.02 |
| --- | --- | --- | --- |

*Citrus grandis*

| SDS/Sephacryl [11] | 0.10 – 0.15 | ND | 1.80 |
| --- | --- | --- | --- |

***Vegetables***

Potato (*Solanum tuberosum* L. cv. Atlantic) *(present study)*

| TENS-CO (present study)  MO BIO PowerPlant Pro Kit (present study)  Synergy Plant DNA Kit (present study)  SDS [8] | 0.50 ± 0.04  0.05 ± 0.004  0.20 ± 0.01  0.50 – 0.80 | 2.23 ± 0.006  2.41 ± 0.05  2.18 ± 0.05  ND | 1.91 ± 0.04  1.94 ± 0.03  1.93 ± 0.02  > 1.77 |
| --- | --- | --- | --- |

Sweet Potato (*Ipomoea batatas* (L.) Lam.)

| Rapid CTAB [12] | 0.97 ± 0.11 | ND | 1.99 ± 0.09 |
| --- | --- | --- | --- |

Tomato (*Solanum lycopersicum* L. cv. Lance) *(present study)*

| TENS-CO (present study)  MO BIO PowerPlant Pro Kit (present study)  Synergy Plant DNA Kit (present study)  SDS/PVP [13]  MPD/PIPES/PVP [14] | 0.66 ± 0.06  0.05 ± 0.004  0.11 ± 0.01  0.05 – 0.08  0.01 | 2.10 ± 0.05  1.87 ± 0.08  1.93 ± 0.03  ND  ND | 1.93 ± 0.01  1.80 ± 0.01  1.81 ± 0.01  1.80 – 1.93  1.80 – 1.93 |
| --- | --- | --- | --- |

Yam (*Dioscorea* spp.)

| SDS [15] | 0.13 – 0.70 | ND | 1.79 – 2.00 |
| --- | --- | --- | --- |

Common Bean (*Phaseolus vulgaris*)

| SDS [8]  SDS/PVP [9] | 0.50 – 0.80  0.02 – 0.10 | ND  ND | > 1.77  1.65 – 1.80 |
| --- | --- | --- | --- |

Faba Bean (*Vicia faba*)

| SDS [8] | 0.50 – 0.80 | ND | > 1.77 |
| --- | --- | --- | --- |

Lettuce (*Lactuca sativa*)

| SDS [8]  SDS/PVP [9] | 0.50 – 0.80  0.02 – 0.10 | ND  ND | > 1.77  1.65 – 1.80 |
| --- | --- | --- | --- |

***Cotton***

*Gossypium arboreum*

| SDS/CTAB/PVP 40 [6] | 0.05 | ND | 1.88 |
| --- | --- | --- | --- |

*G. herbaceum*

| SDS/CTAB/PVP 40 [6] | 0.05 | ND | 2.17 |
| --- | --- | --- | --- |

***Woody Plants***

Grapevine (*Vitis* spp.)

| CTAB/PVP [16]  CTAB/PVP [17]  CTAB/PVP 40 [18] | 0.79 ± 0.14  0.65  0.67 ± 0.01 | ND  1.76  1.93 ± 0.03 | 1.80 – 2.00  1.80  1.98 ± 0.05 |
| --- | --- | --- | --- |

Porcelain-berry *(Ampelopsis brevipedunculata)*

| CTAB/PVP [16] | 0.85 ± 0.05 | ND | 1.80 – 2.00 |
| --- | --- | --- | --- |

Apple (*Malus domestica* cv. Red Delicious)

| CTAB/PVP [16]  CTAB/PVP 40 [18] | 0.83  0.02 ± 0.002 | ND  2.03 ± 0.04 | 1.80 – 2.00  1.90 ± 0.03 |
| --- | --- | --- | --- |

Pear (*Pyrus syrica*)

| SDS [8]  CTAB/PVP 40 [18] | 0.50 – 0.80  0.03 ± 0.003 | ND  1.99 ± 0.01 | > 1.77  1.95 ± 0.04 |
| --- | --- | --- | --- |

Wild Almond (*Prunus amygdalus*)

| SDS [8] | 0.50 – 0.80 | ND | > 1.77 |
| --- | --- | --- | --- |

Coffee (*Coffea brassii*)

| CTAB/PVP [19] | 0.015 – 0.02 | 1.91 | 1.68 |
| --- | --- | --- | --- |

Eucalypt (*Corymbia* *sp*.)

| CTAB/PVP [19] | 0.005 | 1.85 | 1.43 |
| --- | --- | --- | --- |

­­Weeping Fig *(Ficus benjamina)*

| SDS/Sephacryl [11] | 0.10 – 0.15 | ND | 1.80 |
| --- | --- | --- | --- |

CTAB: cetyltrimethylammonium bromide; SDS: sodium dodecyl sulfate; MPD: 2-methyl-2,4-pentanediol; PIPES: piperazine-N,N'-bis(2-ethanesulfonic acid); PVP: polyvinylpyrrolidone; ND: Not determined

**References**

1. Tai TH, Tanksley SD. A rapid and inexpensive method for isolation of total DNA from dehydrated plant tissue. Plant Mol Biol Rep. 1990;8:297–303.
2. Aljanabi SM, Forget L, Dookun A. An improved and rapid protocol for the isolation of polysaccharide- and polyphenol- free sugarcane DNA. Plant Mol Biol Rep. 1999;17:1–8.
3. Bermúdez-Guzmán MJ, Guzmán-González S, Orozco-Santos M, Velázquez-Monreal JJ, Buenrostro-Nava MT, Michel-López CY. Optimizing a protocol for DNA isolation of leaf *Saccharum officinarum.* Rev Mex de Cienc Agríco. 2016;7:897–910.
4. Vaze A, Nerkar G, Pagariya M, Devarumath RM, Prasad DT. Isolation and PCR amplification of genomic DNA from dry leaf samples of sugarcane. Int J Pharma Bio Sci. 2010;1-6.
5. Honeycutt RJ, Sobral BWS, Keim P, Irvine JE. A Rapid DNA extraction method for sugarcane and its relatives. Plant Mol Biol Rep. 1992;10:66–72.
6. Niu C, Kebede H, Auld DL, Woodward JE, Burow G, Wright RJ. A safe inexpensive method to isolate high quality plant and fungal DNA in an open laboratory environment. Afr J Biotechnol. 2008;7:2818–2822.
7. Oard JH, Dronavalli S. Rapid isolation of rice and maize DNA for analysis by random-primer PCR. Plant Mol Biol Rep. 1992;13:236–241.
8. Aljanabi SM, Martinez I. Universal and rapid salt-extraction of high quality genomic DNA for PCR-based techniques. Nucleic Acids Res. 1997;25:4692–4693.
9. Marechal-Drouard L, Guillemaut P (1995) A powerful but simple technique to prepare polysaccharide-free DNA quickly and without phenol extraction. Plant Mol Biol Rep. 1995;13:26–30.
10. Chee PP, Drong RF, Slightom JL. Using polymerase chain reaction to identify transgenic plants. In Gelvin SB, Schilperoort RA, Verma DPS, editors. Plant molecular biology manual. Dordrecht: Springer; 1991. p. 1–28.
11. Li QB, Cai Q, Guy CL. A DNA extraction method for RAPD analysis from plants rich in soluble polysaccharides. Plant Mol Biol Rep. 1994;12:215–220.
12. Kim SH, Hamada T. Rapid and reliable method of extracting DNA and RNA from sweet potato, *Ipomoea batatas* (L.). Lam Biotech Lett. 2005;27:1841–1845.

Peterson DG, Schulze SR, Sciara EB, Lee SA, Bowers JE, Nagel A, Jiang N, Tibbitts DC, Wessler SR, Paterson AH. Integration of Cot analysis, DNA cloning, and high-throughput sequencing facilitates genome characterization and gene discovery. Genome Res. 2002;12:795–807.

1. Peterson DG, Boehm KS, Stack SM. Isolation of milligram quantities of nuclear DNA from tomato (*Lycopersicon esculentum*), a plant containing high levels of polyphenolic compounds. Plant Mol Biol Rep. 1997;15:148–153.

Asemota HN. A fast, simple, and efficient miniscale method for the preparation of DNA from tissues of yam (*Dioscorea* spp.). Plant Mol Biol Report. 1995;13:214–218.

1. Lodhi MA, Ye G-N, Weeden NF, Reisch BI. A simple and efficient method for DNA extraction from grapevine cultivars and *Vitis* species. Plant Mol Biol Rep. 1994;12:6–13.
2. Redzadoost MH, Kordrostami M, Kumleh HH. An efficient protocol for isolation of inhibitor-free nucleic acids even from recalcitrant plants. 3 Biotech. 2016;6:61, doi: 10.1007/s13205-016-0375-0.
3. Japelaghi RH, Haddad R, Garoosi GA. Rapid and efficient isolation of high quality nucleic acids from plant tissues rich in polyphenols and polysaccharides. Mol Biotechnol. 2011;49:129–137.
4. Healey A, Furtado A, Cooper T, Henry RJ. Protocol: a simple method for extracting next-generation sequencing quality genomic DNA from recalcitrant plant species. Plant Methods. 2014;10:21.
